# *Notes from the Field*: Multistate Coccidioidomycosis Outbreak in U.S. Residents Returning from Community Service Trips to Baja California, Mexico — July–August 2018

**DOI:** 10.15585/mmwr.mm6814a5

**Published:** 2019-04-12

**Authors:** Mitsuru Toda, Francisco J. Gonzalez, Maureen Fonseca-Ford, Patrick Franklin, Melinda Huntington-Frazier, Bruce Gutelius, Vance Kawakami, Kristy Lunquest, Stephanie McCracken, Kathleen Moser, Hanna Oltean, Adam J. Ratner, Chelsea Raybern, Kimberly Signs, Allison Zaldivar, Tom M. Chiller, Brendan R. Jackson, Orion McCotter

**Affiliations:** ^1^Epidemic Intelligence Service, CDC; ^2^Mycotic Diseases Branch, Division of Foodborne, Waterborne, and Environmental Disease, National Center for Emerging and Zoonotic Infectious Diseases, CDC; ^3^Departments of Pediatrics and Microbiology, New York University School of Medicine, New York, New York; ^4^United States-Mexico Unit, Division of Global Migration and Quarantine, National Center for Emerging and Zoonotic Infectious Diseases, CDC; ^5^Missouri Department of Health and Senior Services; ^6^Public Health – Seattle & King County, Seattle, Washington; ^7^New York City Department of Health and Mental Hygiene, New York, New York; ^8^Maryland Department of Health; ^9^Michigan Department of Health and Human Services; ^10^Washington State Department of Health; ^11^Kansas Department of Health and Environment.

On August 8, 2018, the New York City Department of Health and Mental Hygiene notified CDC about two high school students hospitalized for pneumonia of unknown etiology who had recently returned from community service trips constructing houses near Tijuana in Baja California, Mexico. Patients had developed fever 9 and 11 days after travel, followed by rash and lower respiratory symptoms. Symptoms did not improve with multiple courses of antibacterial medications, and the patients subsequently received diagnoses of coccidioidomycosis, a fungal disease commonly known as valley fever.

Given the occurrence of severe illness in two young and previously healthy persons, additional case finding was conducted through outreach to the school group and an organization that coordinates service trips, as well as through Epi-X* notices. By October 15, 2018, eight cases of clinically diagnosed valley fever had been reported in four states (Kansas, Maryland, Michigan, and New York) in persons who traveled on multiple service trips during June–July 2018 (Figure). Four patients were hospitalized, including one who required intensive care, one who required chest tube placement for pleural effusion, and one who was hospitalized for >10 days. All patients were male, five were high school students, and three were adults. Patients were part of seven separate trips organized by churches, high schools, or community groups. These trips were coordinated by two separate organizations and involved an estimated 225 travelers from six states (including, in addition to the four states with identified cases, Missouri and Washington). Seven patients had performed excavation or construction on a single house south of Tijuana, suggesting this site was the likely source of exposure for most patients. State and local health departments notified all travelers about their risk for valley fever. In addition, through binational communication mechanisms, local, state, and federal authorities in Mexico were also alerted to the outbreak. No additional cases associated with this outbreak were detected in Mexico.

**FIGURE:**
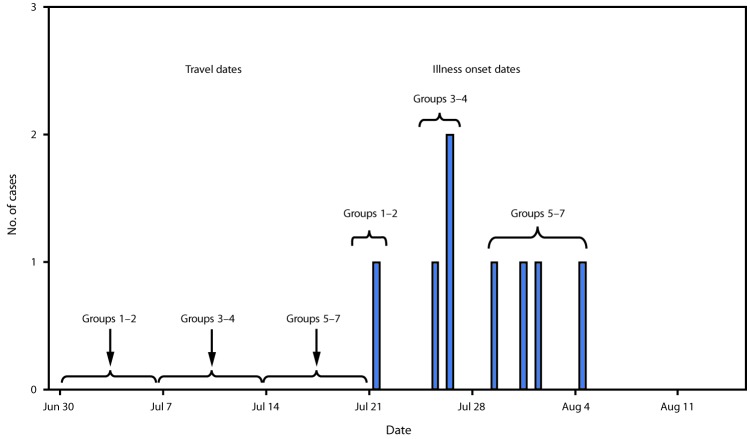
Cases of coccidioidomycosis among U.S. residents returning from community service trips to Baja California, Mexico (N = 8), by date of travel and date of illness onset — Kansas, Maryland, Michigan, and New York, July–August, 2018

Valley fever is primarily acquired through inhalation of airborne dust or soil containing the spores. Approximately 40% of persons develop influenza-like symptoms 1–3 weeks after exposure. Approximately 5%‒10% of persons develop serious pulmonary problems, and an even smaller percent (1%) of persons develop disseminated disease. The disease is endemic in the southwestern United States, northern Mexico, and parts of Central and South America (*1*). In recent years, incidence has increased in California, which borders Baja California (*2*). Valley fever is not a mandatorily reportable disease in Mexico, and standard serological diagnostic testing is generally unavailable, limiting understanding of its epidemiology. Valley fever has been considered endemic in Tijuana but to a lesser extent than in other areas of Mexico (*3*). However, valley fever outbreaks have been reported previously among travelers involved in construction projects, including service trips to the Mexican cities of Tecate (*4*) and Hermosillo (*5*).

The severity of illness and delays in accurate diagnosis observed in this outbreak underscore the importance of obtaining a travel history and considering coccidioidomycosis in persons with respiratory symptoms, with or without rash, who have returned from northern Mexico or areas of the United States where the disease is endemic.^†^ Organizers of service or mission trips involving soil-disturbing activities in these areas should educate participants about the risk for valley fever. Potential mitigation efforts could include soil wetting, employing professionals with appropriate occupational safety training for excavation, staying upwind of digging when possible, and using at minimum CDC’s National Institute for Occupational Safety and Health–approved or Food and Drug Administration–cleared N-95 respirators when performing dust-generating activities. Finally, improved early diagnosis, treatment, and surveillance capacities for valley fever could reduce misdiagnosis, improve patient outcomes, and allow for more targeted public education.

## Footnotes

† https://www.cdc.gov/fungal/diseases/coccidioidomycosis/maps.html.
